# Handedness in Neandertals from the El Sidrón (Asturias, Spain): Evidence from Instrumental Striations with Ontogenetic Inferences

**DOI:** 10.1371/journal.pone.0062797

**Published:** 2013-05-06

**Authors:** Almudena Estalrrich, Antonio Rosas

**Affiliations:** PaleoAnthropology Group, Department of Paleobiology, Museo Nacional de Ciencias Naturales (MNCN-CSIC), Madrid, Spain; Institut de Biologia Evolutiva - Universitat Pompeu Fabra, Spain

## Abstract

The developed cognitive capabilities for *Homo sapiens* seems to be the result of a specialized and lateralized brain, and as a result of this, humans display the highest degree of manual specialization or handedness among the primates. Studies regarding its emergence and distribution within the genus *Homo* show that handedness is present very early. The mode in which it was articulated and spread across the different species during the course of human evolution could provide information about our own cognitive capacities. Here we report the manual laterality attributed to eleven 49,000 old Neandertal individuals from El Sidrón cave (Spain), through the study of instrumental or cultural striations on the anterior dentition. Our results show a predominant pattern addressed to right-handers. These results fit within the modern human handedness distribution pattern and provide indirect evidence for behavior and brain lateralization on Neandertals. They support the early establishment of handedness in our genus. Moreover, the individual identified as Juvenile 1 (6–8 years old at death), displays the same striation pattern as the adult Neandertals from the sample, and thereby the ontogenic development of manual laterality in that Neandertal population seems to be similar to that of living modern humans.

## Introduction

Handedness is the tendency to display left- or right-hand task preference, and modern humans show a high frequency of right hand specialization compared to all great apes, meaning that such pattern of manual laterality should have been developed only in our actual lineage.

In order to understand and define how and when handedness evolved at population-level, several studies have been carried out through the paleoanthropological record employing different methodologies like stone tool production [1]; [2]; [3], the analysis of cut mark orientation on bones [4]; [5], brain lateralization from endocasts [6], asymmetries in hominid upper limb skeletons [7], and the orientation of scratches striating the labial surface of anterior teeth [8].

As a result it is acknowledged that during Middle Pleistocene, *Homo heidelbergensis* had an exclusively right-handed preference [1]; [8]; [9]; [10]; [11]; [12].

For Neandertals, widespread evidence from France to Spain to Croatia, beginning at least 130,000 years ago shows that they followed a right-handed pattern close to that characteristic to modern *Homo sapiens*. Studies by Bermúdez de Castro *et al*. [8], Trinkaus *et al*. [13], Lalueza-Fox and Frayer [14], Frayer *et al*. [15] and Volpato *et al*. [16] on skeletal evidence, indicate that although there is a predominant right-hand use at species-level, at least two individuals with a left-handed preference have been identified, showing a frequency of 93% right-handers to 7% left-handers [16]. Thus, Neandertals show a frequency identical to modern human populations [17]; [18].

This study analyzes the directionality of the instrumental or cultural striations present on the anterior teeth of eleven individuals of known sex and age from El Sidrón cave (Asturias, Spain), substantially increasing the Neandertal sample.

### El Sidrón Cave

The sample comprises teeth from the Neandertal fossils of El Sidrón cave, which are housed at the Department of Paleobiology at National Museum of Natural History (MNCN- CSIC) in Madrid, Spain. The age of the bone assemblage has been estimated at ∼49 kya [19]; [20]. At the moment, the minimum number of individuals (MNI) identified at the site is 13, including seven adults, three adolescents, two juveniles and one infant [21]; [22]. We have genetic profiles for each individual, so we are reasonably certain of the sex, based on presence (or absence) of Y chromosome markers [21]. So, unlike other studies which estimates sex based on size or robusticity, we approached the sex-estimation also from paleogenetics. The combined results show that the sample comprises at least 3 adult females, 3 adult males, 2 adolescent males, and 1 juvenile possible male. For 4 individuals it was not possible to retrieve genetic material and the skeletal evidence was not enough to fairly conclude the estimation. In addition, several human remains show evidence that they were subject to cannibalism [22]; [23].

**Figure 1:**
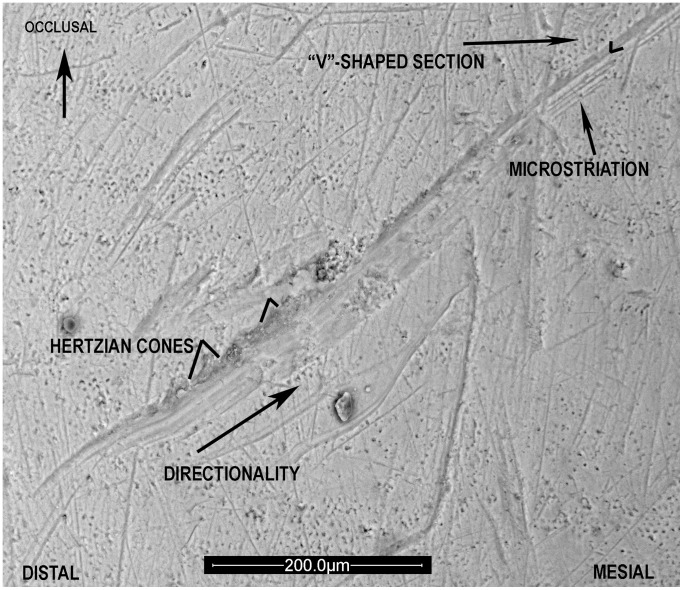
Cultural or Instrumental striation morphology. 200× picture of SD-355 (RI_2_) associated to individual Adult 4. Cut mark features are indicated on the image. Directionality is from cervix to the incisal edge.

The dental fossil sample selected for this study comprises 66 teeth (62 are permanent teeth while 4 are deciduous teeth) corresponding to 11 of the 13 individuals identified at the El Sidrón cave. A complete list of the specimens here analyzed with their catalogue number, anatomical identification and individuals assignment is provided on supplementary materials (Table S1). The individuals infant 1 and juvenile 2 do not have associated teeth. No taphonomical, excavation or preparation damage have been observed on the teeth [22]; [23]. All dental modifications observed on teeth are clearly related to both masticatory and non-mastication wear (see Figure 1 as an example of the good preservation of dental surface). Posterior teeth show no evidence of buccal (or labial) striations.

### Instrumental or Cultural Striations

Instrumental or cultural striations are cut marks associated with the use of teeth as a third hand, known as the “stuff-and-cut” behavior [24]. Brace [24] used this term to describe differential, heavy wear on Neandertal incisors and canines compared to their premolars and molars. When an individual holds some material between the teeth with one hand pulling and the opposite hand cutting the material, scratches are sometimes left on the labial surface of incisors and canines when the lithic tool accidentally glances the tooth surface. Experimental work using teeth from dental extractions [8]; [9] has produced cut marks on enamel similar to those found on original Neandertal teeth.

Several authors have characterized the morphology of cultural striations on Pleistocene hominids ([8]; [9]; [10]; [11]; [14]; [15]; [16], among others). The scratches appear mostly on the labial surface of the central and lateral incisors and are more common in the central area than the mesial or distal sides of the teeth. On the canine they most commonly occur near the mesial surface. Their length varies between 1 to 4 mm and the width varies from 20 to 100 microns. These striations exhibit the same morphological features of the cut marks on bone [4]; [25]; [26], *i.e.*, Hertzian cones, grooves with a “V”- shaped section, and microscratches at the bottom, which vary depending on the stone tool that produced the striation [9], and, at least, linear and parallel borders. Because of this morphology it is possible to establish the directionality of the cut mark [26] and infer the manual laterality of the individual who has made the cut mark.

Based on these features, Bermúdez de Castro *et al.* [8], Lalueza-Fox and Frayer, [14] and Lozano-Ruíz *et al*. [9], linked the orientation and directionality of the cultural striation with a preferred hand movement, leading to the establishment of a direct relation between the individual’s hand movement and the directionality of the cultural striation. Once a statistical significance was reached, it allowed the assignment of handedness to individuals.

Instrumental striations have been reported on Middle and Late Pleistocene fossil hominids from the sites of La Quina [27]; [28], L’Hortus [29]; [8], Cova Negra [8], Shanidar [30], Krapina [14], Vindija [15], Regourdou [16], Tabun I [28], Broken Hill [28], Boxgrove [31] and Sima de los Huesos [8]; [9]; [10]; [12], as well as in several modern human populations, such as the Chalcolithic individuals from Pakistan [32], Neolithic from Spain [33] and Sweden [34], Paleoindian from North America [35], Australian Aborigines [10], and Fueguians [33].

## Methods

Each tooth was inspected under binocular lens and Environmental Scanning Electron Microscope (ESEM Fei- Quanta 200 ^©^ located in the National Museum of Natural History (MNCN-CSIC). All teeth were examined at 25.0 kv accelerating voltage and low vacuum mode. The magnification observations ranged from 40× to 1000× in some instances.

As 21 teeth were found *in situ* (in their respective *alveoli* on both the maxilla and mandible), it has been necessary to perform high-resolution replicas of them, due to the limited dimensions of the microscope’s chamber. Following the procedures described elsewhere in detail, a hydrophilic vinyl polysiloxane resin was employed to make the negative cast [26], and a polyurethane resin to make the replica [36]. Using a low viscosity variety it is possible to produce replicas of great resolution and the details on the negative impression can last more than a year; polyurethane has a moderately fast drying time, excellent viscosity and fluidity that allow faithfully details reproduction of the enamel surface. Finally, the replicas were covered with a thin layer of metallic gold (Sputter Coater, EMITECH K550Y®) to allow the ESEM visualization.

The orientation of the *striae* to the occlusal plane was measured directly on digital images (NIH Image J, Image Processing and Analysis in Java; [37]). In this study, we have considered the instrumental striations that have an undoubted cut-mark morphology, that is: we did not count the scratches that appear worn (cut-mark morphology is almost removed, and thus we cannot be sure about their origin) or the ones that are short and thin (thinner than 20 microns and length less than 1 mm, as was described before according to the results on the Krapina Neandertals sample [14]), and could be confused with dietary dental microwear. Orientation's classification was made following Bermúdez de Castro *et al*. [8]. X^2^ tests were performed to examine the significance (p<0.05) of the striations' distribution on each individual.

## Results

**Figure 2:**
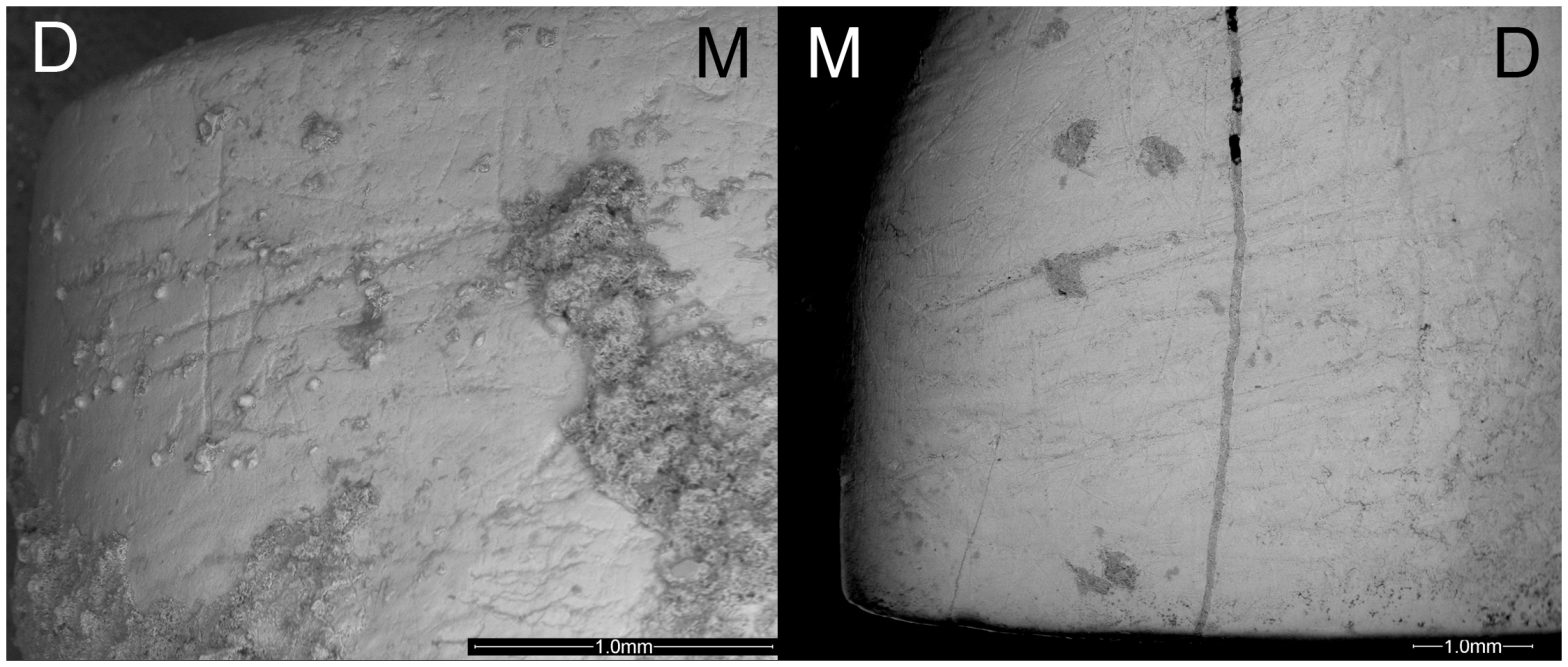
Instrumental striations present on the El Sidrón Adolescents. Left: LI_2_ from Adolescent 1. Right: LI^1^ belonging to Adolescent 2. All instrumental striations are distinguishable, and some are also still slightly covered by sediment, that reinforces their ancient origin. M = mesial; D = distal.

**Figure 3:**
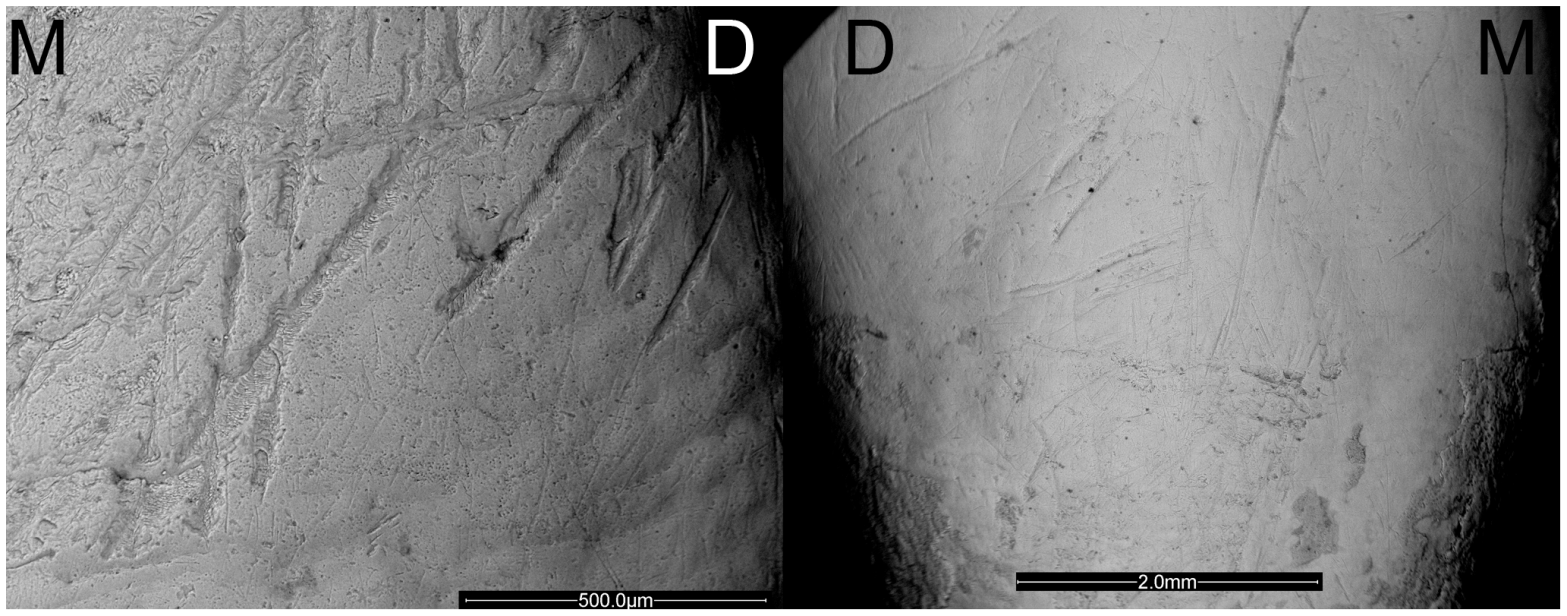
Instrumental striations present on two El Sidrón Adult females. Scanning Electron Microscope images of the instrumental striations identified on the left upper canine on Adult 3 (Left) and on the right lower lateral incisor from Adult 4 (Right). M = mesial; D = distal.

**Figure 4:**
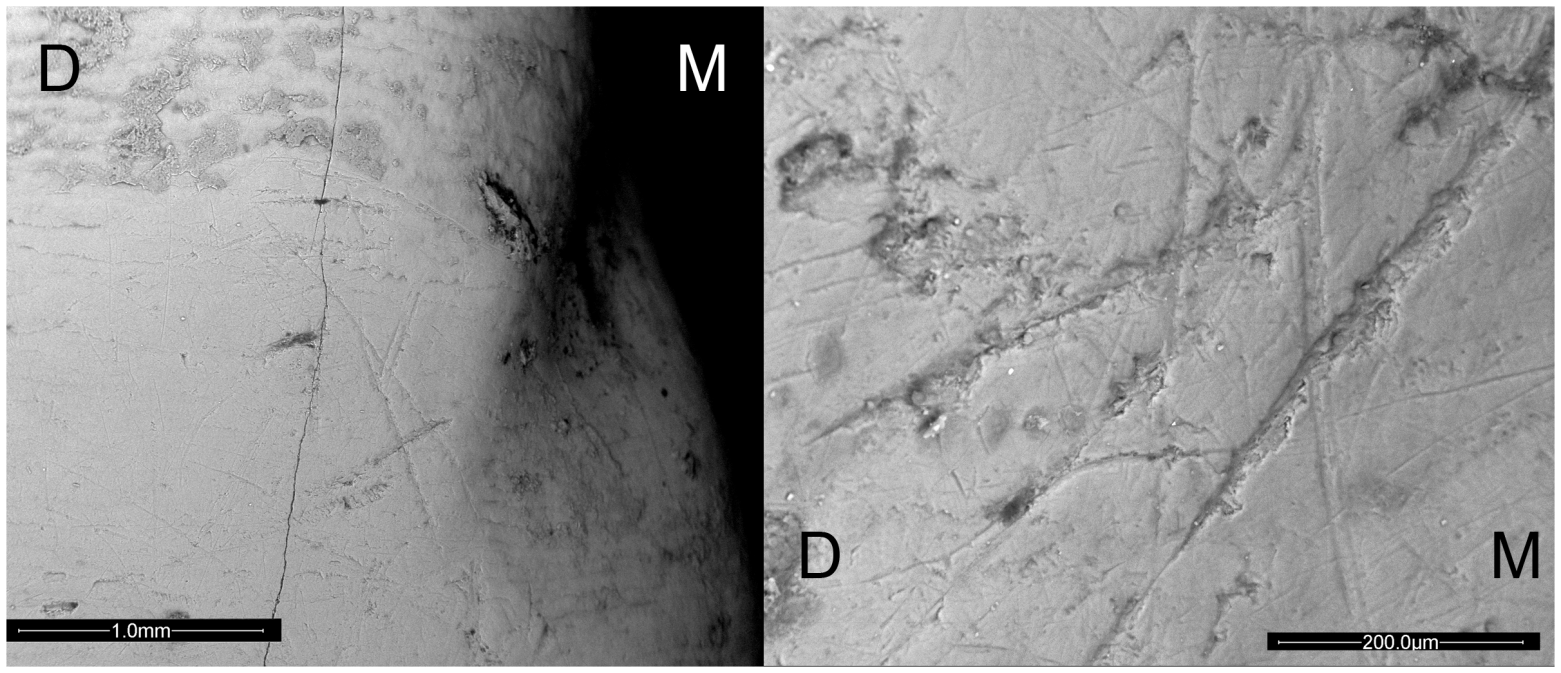
Instrumental striations present on one El Sidrón Adult males. ESEM micrographs of the labial surface of the upper right central incisor and upper right canine belonging to Adult 1. M = mesial; D = distal.

All individuals studied at the El Sidrón site present instrumental striations (Figures 2, 3 and 4). The results of the characterization of the orientation for each tooth associated to an individual are shown in Tables 1 and 2. Considering them as a group, the preferred orientation is right-oblique (58% of the total number of cultural striations analyzed), followed by the horizontal orientation (17% of the total). Vertical (14%) and left-oblique (11%) orientations are also present in all the individuals but their frequencies are not high.

**Table 1:** Instrumental striations on the El Sidrón adult Neandertals.

	MAXILLARY DENTITION						MANDIBULAR DENTITION					
INDIVIDUAL	RC	RI2	RI1	LI1	LI2	LC	RC	RI2	RI1	LI1	LI2	LC
**ADULT 1**	n = 32	n = 19	n = 24	n = 16	n = 20	n = 16	n = 17	n = 13	n = 14		n = 13	n = 15
	RO = 19	RO = 10	RO = 15	RO = 9	RO = 11	RO = 12	RO = 11	RO = 8	RO = 12		RO = 6	RO = 9
	H = 4	H = 3	H = 2	H = 3	H = 3	H = 0	H = 1	H = 2	H = 1		H = 4	H = 4
	LO = 3	LO = 2	LO = 3	LO = 1	LO = 3	LO = 2	LO = 2	LO = 0	LO = 0		LO = 1	LO = 1
	V = 6	V = 4	V = 4	V = 3	V = 3	V = 2	V = 3	V = 3	V = 1		V = 2	V = 1
**ADULT 2**	n = 8	n = 14	n = 15	n = 17	n = 12	n = 16	none	n = 16	n = 16	n = 17	none	none
	RO = 3	RO = 5	RO = 6	RO = 9	RO = 4	RO = 6		RO = 6	RO = 7	RO = 7		
	H = 2	H = 1	H = 0	H = 2	H = 3	H = 2		H = 5	H = 2	H = 4		
	LO = 3	LO = 7	LO = 7	LO = 4	LO = 3	LO = 7		LO = 4	LO = 4	LO = 5		
	V = 0	V = 1	V = 2	V = 2	V = 2	V = 1		V = 1	V = 3	V = 1		
**ADULT 3**	none	none	none	none	none	n = 31	none	n = 17	n = 12	none	none	none
						RO = 23		RO = 8	RO = 8			
						H = 4		H = 3	H = 3			
						LO = 3		LO = 2	LO = 1			
						V = 1		V = 4	V = 0			
**ADULT 4**	n = 15	none	none	n = 31	none	n = 26	n = 23	n = 27	n = 29	n = 29	n = 18	none
	RO = 8			RO = 19		RO = 15	RO = 17	RO = 14	RO = 21	RO = 23	RO = 13	
	H = 2			H = 5		H = 4	H = 3	H = 6	H = 4	H = 4	H = 4	
	LO = 1			LO = 2		LO = 2	LO = 0	LO = 4	LO = 3	LO = 0	LO = 0	
	V = 4			V = 5		V = 5	V = 3	V = 3	V = 1	V = 2	V = 1	
**ADULT 5**	n = 8	n = 18	n = 17	n = 25	n = 22	n = 8	none	n = 15	n = 26	n = 22	n = 22	n = 16
	RO = 7	RO = 11	RO = 11	RO = 17	RO = 13	RO = 5		RO = 7	RO = 23	RO = 9	RO = 13	RO = 7
	H = 0	H = 2	H = 2	H = 3	H = 4	H = 2		H = 3	H = 0	H = 6	H = 6	H = 5
	LO = 0	LO = 2	LO = 0	LO = 2	LO = 3	LO = 0		LO = 1	LO = 1	LO = 2	LO = 1	LO = 1
	V = 1	V = 3	V = 4	V = 3	V = 2	V = 1		V = 4	V = 2	V = 5	V = 2	V = 3
**ADULT 6**	none	none	none	n = 18	none	n = 22	none	none	none	none	n = 21	n = 26
				RO = 11		RO = 15					RO = 8	RO = 11
				H = 3		H = 4					H = 8	H = 9
				LO = 3		LO = 1					LO = 2	LO = 1
				V = 1		V = 2					V = 3	V = 5
**ADULT 7**	none	n = 22	n = 20	none	none	none	none	none	none	none	none	none
		RO = 15	RO = 11									
		H = 3	H = 3									
		LO = 1	LO = 2									
		V = 3	V = 4									

In this table are the results for the analysis of the total number of instrumental striations counted per tooth (n) and the number of scratches depending on their orientation. RC: right canine; RI2: right lateral incisor; RI1: right central incisor; LI1: left central incisor; LI2: left lateral incisor; LC: left canine. RO: right-oblique orientation; LO: left-oblique orientation; V: vertical orientation; H: horizontal orientation; n:.

**Table 2:** Instrumental striations on the El Sidrón immature Neandertals.

	MAXILLARY DENTITION						MANDIBULAR DENTITION					
INDIVIDUAL	RC	RI2	RI1	LI1	LI2	LC	RC	RI2	RI1	LI1	LI2	LC
**ADOLESCENT 1**	n = 10	none	none	none	none	none	none	n = 13	n = 27	none	none	none
	RO = 7							RO = 6	RO = 16			
	H = 1							H = 2	H = 3			
	LO = 1							LO = 1	LO = 2			
	V = 1							V = 4	V = 6			
**ADOLESCENT 2**	none	n = 39	n = 28	n = 24	none	none	none	n = 16	none	none	none	none
		RO = 27	RO = 18	RO = 12				RO = 9				
		H = 6	H = 3	H = 7				H = 3				
		LO = 2	LO = 3	LO = 3				LO = 4				
		V = 4	V = 4	V = 2				V = 0				
**ADOLESCENT 3**	none	none	none	n = 15	n = 15	n = 16	none	none	none	none	none	none
				RO = 7	RO = 9	RO = 14						
				H = 3	H = 0	H = 2						
				LO = 4	LO = 1	LO = 0						
				V = 1	V = 5	V = O						
**JUVENILE 1**	n = 6*	none	n = 16	n = 20	none	none	n = 14*	n = 18*	n = 16	n = 19	none	n = 7*
	RO = 4		RO = 4	RO = 7			RO = 8	RO = 9	RO = 12	RO = 9		RO = 3
	H = 1		H = 5	H = 4			H = 2	H = 2	H = 3	H = 2		H = 1
	LO = 0		LO = 2	LO = 3			LO = 1	LO = 0	LO = 1	LO = 3		LO = 2
	V = 1		V = 5	V = 6			V = 3	V = 7	V = 0	V = 5		V = 1

The results for the analysis of the number of instrumental striations (n) counted on each tooth and the number according to their orientation through the occlusal/incisal plane on the El Sidrón adolescent and juvenile Neandertals. * Indicate deciduous teeth. RC: right canine; RI2: right lateral incisor; RI1: right central incisor; LI1: left central incisor; LI2: left lateral incisor; LC: left canine. RO: right-oblique orientation; LO: left-oblique orientation; V: vertical orientation; H: horizontal orientation.

The tooth with the highest number of cultural striations registered in the sample belongs to adolescent 2, followed by adult 1 and adults 3 and 4. Regarding the strations' measurements on the sample, the mean length is 2.063 mm (SD = 0.147) and the mean width is 43.5 microns (SD = 16 microns), similar to the Krapina Neandertals [14].

**Figure 5:**
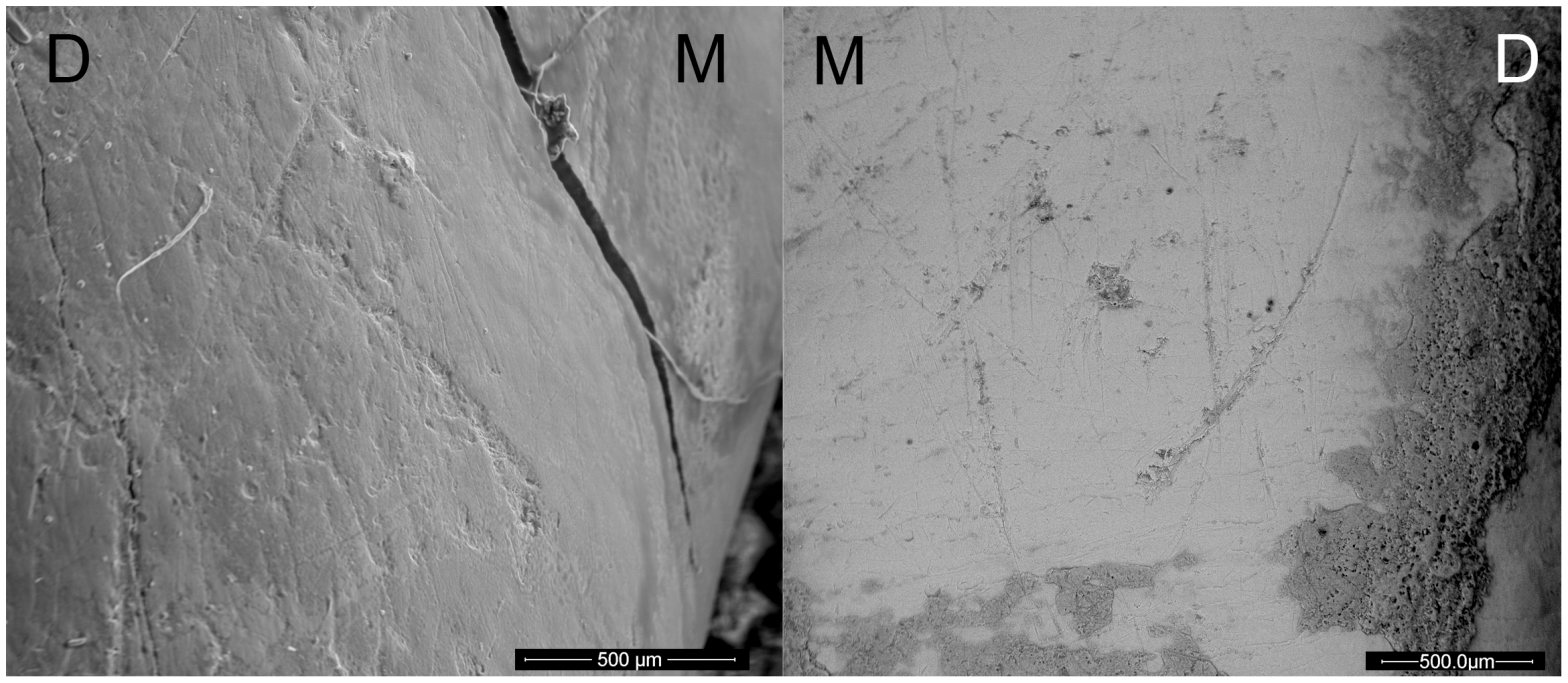
Instrumental striations on one El Sidrón Adult and the juvenile Neandertal. Morphological comparison between the instrumental striations found on: Left: a mandibular right deciduous lateral incisor (SD-1600c) from Juvenile 1; and Right: a mandibular right lateral incisor belonging to Adult 5 (SD-1327a). M = mesial; D = distal.

Juvenile 1 has several instrumental striations, whose morphology has been carefully studied, and coincide with the cultural striations pattern, like the adult Neandertals from the sample, as shown in Figure 5.

The X^2^ analyses comparing the number of right vs left scratches reveal that all X^2^ values exceed the table value of 7.815 (at 3 degrees of freedom and p<0.00001). Based on this we can reject the null hypothesis of a random distribution in favor of the hypothesis the striations are showing a preferred orientation on every El Sidrón Neandertal (Table 3), which is right-oblique.

**Table 3:** Comparison of number of right vs left scratches.

Individual	Chi-square	df	p
Adult 1	70.16	3	<.0000001
Adult 2	24.48	3	<.0000001
Adult 3	87.12	3	<.0000001
Adult 4	91.68	3	<.0000001
Adult 5	75.76	3	<.0000001
Adult 6	46.64	3	<.0000001
Adult 7	75.12	3	<.0000001
Adolescent 1	62.24	3	<.0000001
Adolescent 2	74.80	3	<.0000001
Adolescent 3	85.44	3	<.0000001
Juvenile 1	34.60	3	<.0000001

The results of the X^2^ analysis (x^2^ values exceed the table value of 7.815 at 3 degree of freedom) show that all values accept a preferred orientation regarding the instrumental striations' distribution, and in this sample is oblique to the right.

## Discussion

As said, all individuals analyzed from the El Sidrón individuals have cut marks on their erupted anterior dentition. Our results show a predominantly right-oblique orientation of the instrumental striations, typically ascribed to right-handers [14]. The results match with the modern human handedness pattern, and provide additional indirect evidence for Neandertal brain lateralization. This handedness pattern, together with the asymmetries on cranial dural sinuses and blood drainage [38], the morphology of hyoid bone [39] and the same derived FOXP2 variant of modern humans [40] as part of the basis for human speech capability, reinforces a modern brain asymmetry pattern in Neandertals.

In our sample we found a substantial number of vertical striations (177 out of 1233, or 14% of the total striations counted). The vertical marks have been associated with the processing of plant fibers in Paleoindian populations [41] and according to the authors, it is not possible to address a manual preference on the sample they studied. Since the marks described by Bax and Ungar [41] display the characteristic cut mark morphology associated to the instrumental striations here considered, and are also shorter and thinner, we consider that the behavior responsible for the scratch formation is different in both samples. Therefore, it is not recommended to analyze all the anterior scratches to address handedness in past populations.

**Figure 6:**
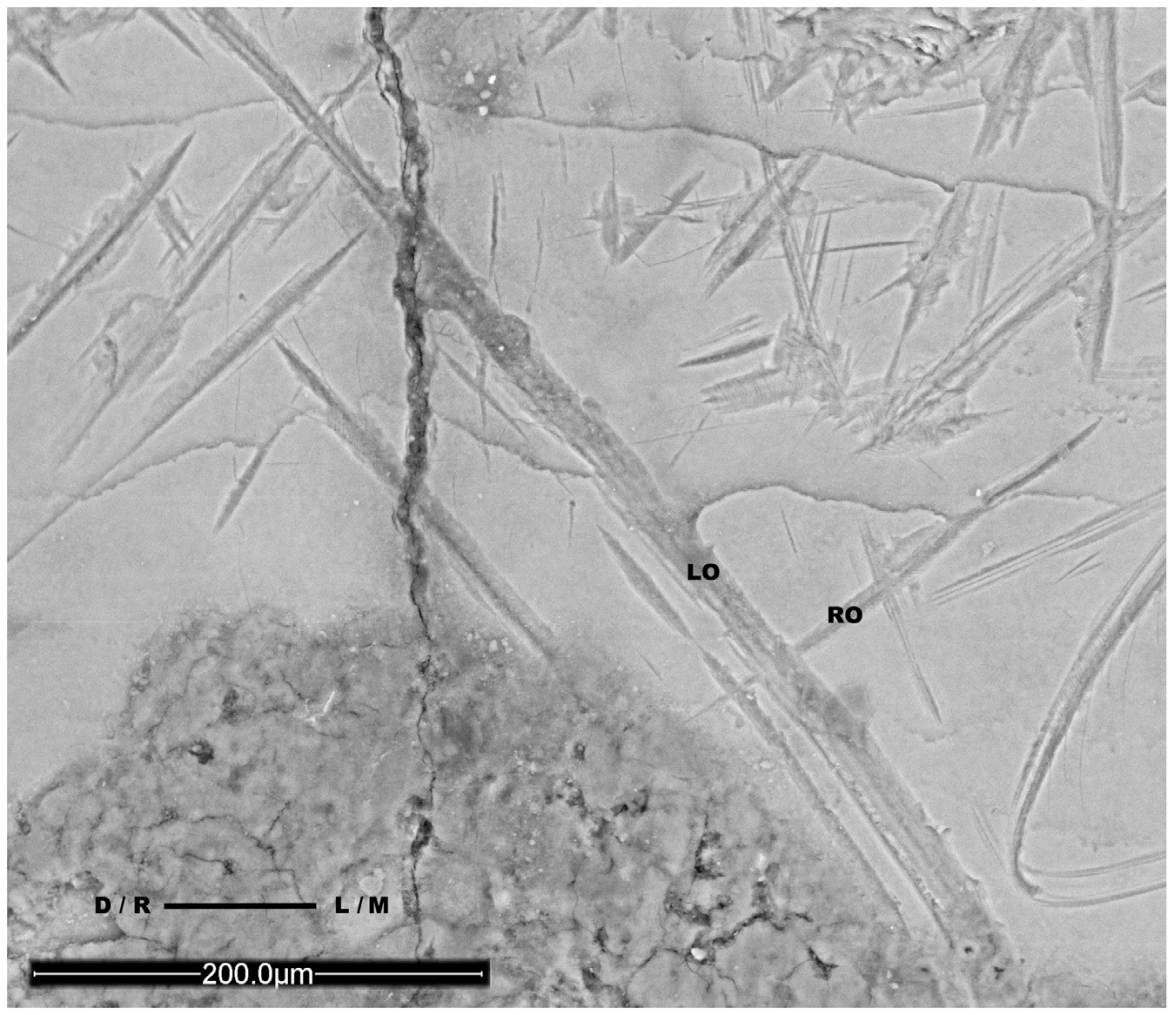
The peculiar case found on El Sidrón Adult 2. ESEM image showing the left-oblique striations covering the right-oblique on a RI_1_ from Adult 2. M = mesial; D = distal. R = right; L =  left.

Individual adult 2 presents almost the same number of right and left-oblique striae, but the left ones are covering the right oriented striations. This pattern appears on both mandibular and maxillary dentition, but is more evident on the lower teeth (Fig. 6). Despite this, the interpretation of X^2^ results show that this individual is right-handed. The presence of a severe oral pathology on the left side on his mandible [42] plus dental calculus deposits on the occlusal molar surface, suggest that this individual may have changed his hand orientation to avoid the pain on his mouth, and can explain the fact that the left-oriented striations appear new when compared with the right ones. We propose that chewing with the left and even the action of holding materials for processing could be painful. That could have motivated the individual to start using the right side of his teeth to seize materials and later cut them, adapting his manual laterality to his actual circumstances. According to the well-preserved striae's morphology (Figure 6), this change may have happened fast, and since no postcranial elements have been associated to this individual yet, we propose the pathological condition as the most plausible explanation.

Juvenile 1 displays the same striation pattern as the other adult Neandertals from the sample, although the number of scratches is slightly below the adults. The number of scratches varies depending on how many times the individual have repeated the action [10]. So, despite the juvenile seems to have performed this task less often than adults, the number indicates that it could be an habitual behavior, and he left enough cut marks on his front teeth to describe him as a right-handed individual.

Previous studies dealing with those scratches were done on adult Neandertals, so this is the first time that handedness has been found in a juvenile Neandertal. The estimated age for this individual is about 6–8 years old, so it seems that the ontogenic development of manual laterality in that Neandertal population is similar to that of actual modern humans. In this specie an unimanual hand use for reaching objects has been detected early on infants [43], although manual laterality preference when performing complex tasks and bimanual actions appear on later age [44] and it is completely established around seven years of age [45].

Bermúdez de *Castro et al*. [8] and Lozano *et al*. [10] presented results of handedness for subadult individuals (mostly adolescents) for the SH sample. On their results, they show evidence for right-handedness on adolescent individuals, and even on one individual with an estimated age at death of 3–4 years old. This individual has only 2 cut marks on one tooth, so we are not sure if it could indicate an incipient establishment of handedness or maybe its number is underrepresented because the individual's anterior dentition has not been recovered.

According to our results, the El Sidrón individuals are all right-handed, and when comparing to other Neandertals described in the literature, the distribution of manual laterality coincides with the handedness distribution at the Vindija cave and Atapuerca-Sima de los Huesos site, whose population appears to consist exclusively of right-handed individuals [9]; [10]; [12]. At the Neandertal sites of Hortus [29] and Krapina [14] the dominant trend is the same, oblique to the right, but two specimens (KDP 4 and Hortus VII) have an oblique to the left predominant orientation, which is characteristics of left-handed individuals. Table 4 summarizes the findings regarding manual lateralization in Neandertals by the study of cultural striations, including the last findings at the El Sidrón fossils. At population level in Neandertals, the distribution of handedness based on this feature is 27 right-handers to 2 left-handers, or 93% to 7% [16]; [18].

**Table 4:** List of Neanderthal sites where handedness has been identified from cultural striations.

INDIVIDUAL	HANDEDNESS	REFERENCES
Cova Negra	R	Bermúdez de Castro *et al*. 1988
Hortus VII	L	de Lumley, 1973; Bermúdez de Castro *et al*. 1988
Hortus VIII	R	de Lumley, 1973; Bermúdez de Castro *et al*. 1988
Hortus IX	R	de Lumley, 1973; Bermúdez de Castro *et al*. 1988
Hortus XI	R	de Lumley, 1973; Bermúdez de Castro *et al*. 1988
Hortus XII	R	de Lumley, 1973; Bermúdez de Castro *et al*. 1988
KDP 4	L	Lalueza-Fox & Frayer, 1997
KDP 5	R	Lalueza-Fox & Frayer, 1997
KDP 6	R	Lalueza-Fox & Frayer, 1997
KDP 17	R	Lalueza-Fox & Frayer, 1997
KDP 18	R	Lalueza-Fox & Frayer, 1997
KDP 29	R	Lalueza-Fox & Frayer, 1997
KDP Q	R	Lalueza-Fox & Frayer, 1997
Le Régourdou 1	R*	Volpato *et al*., 2012
Vindija 206	R	Frayer *et al*., 2010
Vindija 288	R	Frayer *et al*., 2010
Vindija 289	R	Frayer *et al*., 2010
Vindija 290	R	Frayer *et al*., 2010
Sidrón Adult 1	R	This study
Sidrón Adult 2	R	This study
Sidrón Adult 3	R	This study
Sidrón Adult 4	R	This study
Sidrón Adult 5	R	This study
Sidrón Adult 6	R	This study
Sidrón Adult 7	R	This study
Sidrón Adolescent 1	R	This study
Sidrón Adolescent 2	R	This study
Sidrón Adolescent 3	R	This study
Sidrón Juvenile 1	R	This study

On the left column are the specimens that have been studied, and the references of the study (column on the right). The identification as a right- (R) or left-hander (L) is on the central column. KDP: Krapina Dental Person [47]. This table extends the data provided by Uomini [18], considering only data from the analysis of labial scratches. * =  data also from humeral morphology.

This is consistent with the distribution of manual dexterity in modern humans, which in any population, left-handed individuals are expected to be between 3% and 25% of the total, while right-handed individuals could be between 97% and 75% of the population [17]. This is a highly variable range, but indicates that the most probable manual laterality in one population is the right-handed one [17]. In our case, all individuals identified match with right-handed individuals, as was expected in a human population.

Regarding the behavioral consequences derived from the manual laterality, a recent model proposed by Abrams and Panaggio [46], states that the high prevalence of the same manual laterality pattern at the populational level, may suggest a collaborative society with low level of intra-society violence. This was the first model to make a clear correlation between population-level handedness and social behavior and, if we apply its conclusions to the extreme frequency of right-handedness in Neandertals it may suggest a cooperative society, or at least among groups such as the El Sidrón group.

### Conclusions

Individuals identified from the El Sidrón dental sample present a predominant right-oblique orientation of their instrumental striations, typically related to right-handed individuals. The results enhance the knowledge of Neandertal manual laterality and provide additional indirect evidence for Neandertal brain lateralization.

One individual, identified as adult 2, presents evidence for a change in his manual motion, probably as a result to avoid pain on his unhealthy mouth. This could suggest a rapid adaptative behavior in this group.

The handedness' distribution in Neandertals and its ontogenetic development match within the modern human laterality pattern at population level, and may have behavioral and social applications to this fossil species. In addition, those results are giving information about the evolutionary development and establishment of our own asymmetric hand use, indicating that the achievement of this pattern may be shared with our common ancestor.

## Supporting Information

Table S1 **List of the El Sidrón specimens analyzed in this study, with their catalogue number, anatomical identification and individuals assignment.**
(PDF)
