# Supplementary material for: Handedness in Neandertals from the El Sidrón (Asturias, Spain): Evidence from Instrumental Striations with Ontogenetic Inferences
Source: PLoS One. 2013 May 6;8(5):e62797. doi: 10.1371/journal.pone.0062797 (PMC3646041; doi:10.1371/journal.pone.0062797)
Supplement: Table S1 — List of the El Sidrón specimens analyzed in this study, with their catalogue number, anatomical identification and individuals assignment. (PDF) [file pone.0062797.s001.pdf]

Table S1. List of the El Sidrón specimens analyzed in this study. Which includes their catalogue number, anatomical identification and individuals' assignment.

INDIVIDUAL	INVENTORY NUMBER	ANATOMICAL IDENTIFICATION
ADULT 1	SD-1202	ULC
MALE	SD-441	ULI2
	SD-1201	ULI1
	SD-313	URI1
	SDR-141	URI2
	SD-1010	URC
	SDR-006b	LLC
	SDR-006c	LRI1
	SDR-006d	LRI2
	SDR-006e	LRC
	SD-299a	LLI2
ADULT 2	SD-1427g	ULC
MALE	SD-1427h	ULI2
	SD-1427i	URC
	SD-1240	URI2
	SD-1439	ULI1
	SD-1422	URI1
	SDR-007a	LRI2
	SDR-014	LLI2
	SD-703	LRI1
ADULT 3	SD-1220	ULC
FEMALE	SD-1222	LRI2
	SD-1217b	LRI1
ADULT 4	SD-1661	URC
FEMALE	SD-1572	ULI1

	SD-1018	ULC
	SD-209	LRC
	SD-355	LRI2
	SD-599a	LRI1
	SD-599b	LLI1
	SD-757	LLI2
ADULT 5	SD-2010g	ULC
FEMALE	SD-2010h	ULI2
	SD-2010i	ULI1
	SD-2010j	URI1
	SD-2010k	URI2
	SD-2010l	URC
	SD-1327a	LRI2
	SD-1327b	LRI1
	SD-1327c	LLI1
	SD-1327d	LLI2
	SD-1327e	LLC
ADULT 6	SD-311	ULI1
MALE	SD-1161	ULC
	SD-753	LLC
	SD-1574	LLI2
ADULT 7	SD-1707	URI2
UNKNOWN	SD-582	URI1
ADOLESCENT 1	SD-913	ULC
MALE	SD-1019	LRI2
	SD-278	LRI1
ADOLESCENT 2	SDR-013	URI2
UNKNOWN	SD-331a	URI1
	SD-331b	ULI1

	SD-370b	LRI2
ADOLESCENT 3<br>MALE	SD-568	ULI2
	SD-277	ULI1
	SD-1107	ULC
JUVENILE 1<br>MALE	SD-1721	dURC
	SD-1875	URI1
	SD-1719	ULI1
	SD-1600e	dLLC
	SD-1660e	dLRC
	SD-1660f	LRI1
	SD-1716	dLRI2
	SD-322	LLI1
